# ASO Author Reflections: The Prognostic Value of Time to Metastasis in Colorectal Liver Metastasis

**DOI:** 10.1245/s10434-019-07958-9

**Published:** 2019-10-23

**Authors:** Diederik J. Höppener, Cornelis Verhoef

**Affiliations:** Department of Surgical Oncology and Gastrointestinal Surgery, Erasmus MC Cancer Institute, Rotterdam, The Netherlands

## Past

Within the field of colorectal cancer (CRC), especially concerning colorectal liver metastases (CRLM), time to metastasis is generally seen as an important prognostic indicator. The interval between resection of the primary malignancy and the detection of CRLM, also known as the disease-free interval (DFI), is a key element in the current clinical risk scores.^1^,^2^ A recent analysis questioned this, when no noticeable prognostic information was found for the time to metastases in patients with metastatic CRC.^3^ However, no distinction was made between dissemination site(s) and local, systemic, or palliative therapy. This study therefore evaluated the prognostic value of the DFI specifically in patients with CRLM eligible for surgical therapy.

## Present

Our results indeed show that the prognostic value of the DFI in patients eligible for surgical treatment of CRLM is more limited than previously thought.^4^ The DFI proved only prognostic for early disease recurrence, but this earlier disease recurrence did not seem to translate into an impaired overall survival. While this could be due to a higher rate of salvage therapy, no correlation was found between DFI and salvageability for recurrences.

## Future

The results of the study show that prognosis prediction based solely on clinical variables leaves much to be desired, because the clinical variable assessed only proved of limited prognostic value. While clinical variables can sometimes be considered as a proxy for tumor biology, future efforts to improve prediction modelling should perhaps be based more on actual biological factors instead. Potential candidates within the field of CRLM include (but are not limited to) the histopathological growth patterns and the quantification of immune infiltration at the tumour microenvironment.^5^,^6^
